# Addition of Fructooligosaccharides and Dried Plum to Soy-Based Diets Reverses Bone Loss in the Ovariectomized Rat

**DOI:** 10.1093/ecam/nen050

**Published:** 2011-02-20

**Authors:** Catherine D. Johnson, Edralin A. Lucas, Shirin Hooshmand, Sara Campbell, Mohammed P. Akhter, Bahram H. Arjmandi

**Affiliations:** ^1^Research and Development, Abbott Nutrition, Adult Nutrition Research, Development & Scientific Affairs, Columbus, OH 43219, USA; ^2^Department of Nutritional Sciences, Oklahoma State University, Stillwater, OK 74078, USA; ^3^Department of Nutrition, Food & Exercise Sciences, 436 Sandels Building, Florida State University, Tallahassee, FL 32306, USA; ^4^Osteoporosis Research Center, Creighton University, Omaha, NE 68131, USA

## Abstract

Dietary bioactive components that play a role in improving skeletal health have received considerable attention in complementary and alternative medicine practices as a result of their increased efficacy to combat chronic diseases. The objectives of this study were to evaluate the additive or synergistic effects of dried plum and fructooligosaccharides (FOS) and to determine whether dried plum and FOS or their combination in a soy protein-based diet can restore bone mass in ovarian hormone deficient rats. For this purpose, 72 3-month-old female Sprague-Dawley rats were divided into six groups (*n* = 12) and either ovariectomized (Ovx, five groups) or sham-operated (sham, one group). The rats were maintained on a semipurified standard diet for 45 days after surgery to establish bone loss. Thereafter, the rats were placed on one of the following dietary treatments for 60 days: casein-based diet (Sham and Ovx), soy-based diet (Ovx + soy) or soy-based diet with dried plum (Ovx + soy + plum), FOS (Ovx + soy + FOS) and combination of dried plum and FOS (Ovx + soy + plum + FOS). Soy protein in combination with the test compounds significantly improved whole-body bone mineral density (BMD). All test compounds in combination with soy protein significantly increased femoral BMD but the combination of soy protein, dried plum and FOS had the most pronounced effect in increasing lumbar BMD. Similarly, all of the test compounds increased ultimate load, indicating improved biomechanical properties. The positive effects of these test compounds on bone may be due to their ability to modulate bone resorption and formation, as shown by suppressed urinary deoxypyridinoline excretion and enhanced alkaline phosphatase activity.

## 1. Introduction

The postmenopausal period typically occupies one-third of a woman's life [1], with more than 45.6 million women in the United States in the postmenopausal phase [2]. As the demographic shift to a more aged population continues, a growing number of people will be afflicted with osteoporosis [3]. Currently, pharmaceutical therapies, for example, bisphosphonates, calcitonin and parathyroid hormone, have low long-term compliance and are cost prohibitive [4, 5]. Therefore, further exploration of alternatives and/or adjunctive approaches that can produce clinically relevant bone sparing effects would be of interest. Complementary and alternative medicine (CAM) strategies including biologically based practices such as botanicals and prebiotics have recently become more mainstream due to suggestions by conventional health care providers and consumer belief that CAM treatments will be more effective in combating their health care problem compared with prescription drugs.

Conventional health care providers often administer several medications focusing on the pathogenic process of osteoporosis rather than seeking alternatives that maximize the inherent healing ability of a person's body. As a result, considerable interest has been generated recently by the desire of postmenopausal women to find more natural ways to combat osteoporosis. Scientists have turned to explore the health benefits of plant-based bioactive compounds because they contain vitamins and minerals that are essential to bone health and a number of synthetic and natural phytochemicals that have been shown to positively influence bone metabolism. For instance, soy protein and its isoflavones have been shown to improve bone mass in both animal models of osteoporosis [6–8] and pre- and post-menopausal women [9–11]. Studies from our laboratory have demonstrated that dried plum effectively prevents and/or reverses bone loss in rat models of osteoporosis [12–14] and positively influences biomarkers of bone metabolismin postmenopausal women [15]. Recently, we have reported that fructooligosaccharide (FOS), a prebiotic, reverses bone loss in ovariectomized rats [16]. The positive results of our previous work warrant examination of whether a combination of these bioactive components, for example, combining soy with dried plum or soy with both dried plum and FOS, provides a synergistic effect. The goal of the present study was to investigate the extent to which the combination of various bioactive compounds reverses bone loss in an ovariectomized rat model of osteoporosis.

## 2. Methods

### 2.1. Animals and Diets

Seventy-two female, 3-month-old Sprague–Dawley rats (Harlan Sprague-Dawley Inc., Indianapolis, IN) were individually housed in an environmentally controlled facility. Guidelines for the ethical care and treatment of animals from the Animal Care and Use Committee at Oklahoma State University were strictly followed. After 5 days of acclimation, the rats were divided into six groups (*n* = 12/group) and either ovariectomized (Ovx, 5 groups) or sham-operated (sham, 1 group). Prior to surgery, whole-body bone mineral content (BMC) and bone mineral density (BMD) were assessed using dual-energy X-ray absorptiometry (DXA; QDR4500A Elite model, Hologic Inc., Bedford, MA). Thereafter, rats were maintained on AIN-93M (Research Diets, New Brunswick, NJ), a semipurified, powdered casein-based diet, for 45 days to induce bone loss. After bone loss was confirmed, the sham group and one Ovx group continued to receive the same casein-based diet and served as controls. The remaining four Ovx groups were fed the following diet: (i) Ovx + soy; (ii) soy + 7.5% dried plum, (iii) soy + 5% FOS and (iv) soy + 7.5% dried plum + 5% FOS. All diets were adjusted to have equivalent amount of carbohydrate, protein, fat, fiber, total energy, calcium and phosphorus; furthermore, in the soy treatment group, casein was replaced with soy protein. Rats were pair-fed to the average food intake of the sham group and had free access to deionized water. Food intake was recorded every 3 days, and body weight was measured weekly.

### 2.2. Animal Necropsy and Processing of Samples

One day prior to necropsy, rats were placed in metabolic cages and urine was collected. At the end of the 60 day treatment period, rats were anesthetized with a mixture of ketamine and xylazine (100 and 5 mg kg^−1^ body weight, resp.) and bled from the abdominal aorta. Blood samples were collected, and serum was separated by centrifugation at 1500 g for 20 min at 4°C. Aliquots of serum and urine were frozen and kept at −20°C for later analyses. The femurs, tibiae and fourth lumbar vertebrae were removed, cleaned and freed of surrounding soft tissues. The left tibiae were stored in 70% ethanol for histomorphometric analyses while the other bones were frozen at −20°C until analyses. Uteri were collected, blotted and weighed to confirm the success of ovariectomy.

### 2.3. Biochemical Analyses of Serum and Urine

A commercially available radioimmunoassay kit was used to measure serum 17 *β*-estradiol (E_2_) (Diagnostic Products Corp., Los Angeles, CA). The intra- and interassay coefficient of variation (CV) was 3.9 and 4.9% for E_2_. Serum total alkaline phosphatase (ALP) activity and urinary creatinine were determined colorimetrically using commercially available kits from Roche Diagnostics (Branchburg, NJ). These colorimetric tests were performed on a Cobas-Fara II Clinical Analyzer (Montclair, NJ). Urinary deoxypyridinoline (Dpd) was measured by competitive enzyme immunoassay in a microassay stripwell format (Quidel Corporation, Mountain View, CA). The intra- and interassay CVs were 1.9 and 2.8%, 1.7 and 6.3% and 4.3 and 4.6% for ALP, creatinine and Dpd, respectively.

### 2.4. Bone Mineral Density (BMD) and Mineral Content (BMC)

BMD and BMC of the whole body, right femurs and fourth lumbar vertebrae were assessed by DXA equipped with appropriate software for use with small laboratory animals and isolated bones. Whole-body BMD and BMC were assessed the day of the surgery, 45 days after surgery to assure that bone loss has occurred due to ovariectomy and at the end of 60 days of dietary treatment for evaluating treatment effects. To ash the bones, they were dried, weighed and placed in a muffle furnace at 600°C for 24 h. For assessing total mineral content, the amount of ash was weighed and values were reported as percent ash.

### 2.5. Biomechanical Testing of the Left Femur

Femoral strength was assessed by three-point bending performed on a material testing system (Instron 5543, Canton, MA). The femur was placed in a three-point bending fixture such that the posterior surface rests on the lower supports and the upper support touches the anterior surface. One lower support was placed touching the posterior surface at the distal end of the femur, at the point where the metaphysis begins to widen, 5–8 mm from the distal end. During this test, the anterior surface was under compression and the posterior surface was under tension. An initial load (*∼*10 N) was applied to stabilize the specimen. The three-point bending test was performed at a displacement rate of 3 mm min^−1^. The load–displacement curve was recorded simultaneously during the test.

After the mechanical test, the cross-sectional surface at the fracture site was prepared flat for tracing and subsequent inertial and area calculations. The ultimate load, yield load and the stiffness of the specimen were measured from the load-displacement curve. Ultimate stress, yield stress and modulus of elasticity of the specimen were calculated using beam-bending theory using values for second moment of area.

### 2.6. Bone Histomorphometry

At 8 days and at 1 day prior to necropsy, rats were subcutaneously injected with calcein (8 mg kg^−1^ body weight; Sigma, St Louis, MO) for double-flurochrome labeling of bone to determine active mineralization sites and rate of bone formation using histomorphometry. Quantitative histomorphometry of the left tibia was used to examine the dynamics of the bone changes and bone cell activities due to ovariectomy and dietary treatments.

The tibiae were cut at 1 mm distal to the tibio-ibula junction (TFJ) and 19 mm proximal to the TFJ to obtain a proximal portion for cancellous bone histomorphometry. Both the central diaphysis and proximal tibia were placed in Villanueva stain for 72 h and then returned to 70% ethanol. During the next 14 days, the specimens were dehydrated in graded ethanol and acetone and then embedded individually in modified methyl methacrylate. Sections were analyzed with a light/epifluorescent microscope and a video camera interfaced with the BIOQUANT TCW software (R&M Biometrics, Nashville, TN).

## 3. Statistical Analysis

Data analyses involved computation of means and SEM for each of the treatment groups using SAS (Version 8.2, SAS Institute, Cary, NC). Analysis of variance and least square means were calculated using the general linear model procedure and the means were compared using Fisher's least significant difference for comparing groups. Differences were considered significant at *P* < .05.

## 4. Results

### 4.1. Food Intake, Body and Uterine Weights

The success of the surgical procedure was confirmed as the rats in Ovx groups experienced atrophy of uterine tissue (Table 1). Rats in various treatment groups started with similar body weight and were pair-fed to the average food intake of the Sham group throughout the course of the study. However, in spite of pair feeding, the final mean body weight of rats in all Ovx groups was significantly higher than that in the Sham group.

### 4.2. Whole-Body BMD and BMC

**Figure 1:**
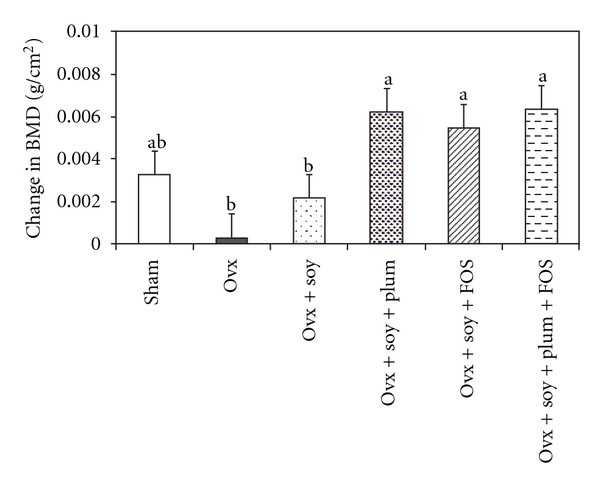
Effects of ovariectomy (Ovx) and various dietary treatments on change in whole-body bone mineral density (BMD). Bars represent mean + SE; *n* = 12 rats per group. Bars with different letters are significantly (*P* < .05) different from each other.

Whole-body BMD and BMC prior to Ovx were not different among the groups (data not shown). However, 45 days after ovariectomy, all Ovx rats on average had 5% lower (*P* < .05) whole-body BMD compared with the sham rats (data not shown). After 60 days of dietary treatment, Ovx rats that were fed soy protein combined with FOS alone (Ovx + soy + FOS) or in combination with dried plum (Ovx + soy + plum + FOS) had significantly higher whole-body BMD when compared with casein-fed Ovx rats, although not quite to the level of the Sham rats (Table 2). Soy protein alone (Ovx + soy) or in combination with dried plum (Ovx + soy + plum) did not improve whole-body BMD (Table 2). When we calculated the change in whole-body BMD (difference in BMD values before and after dietary treatment), soy protein diet in combination with any of the bioactive compounds caused a significantly higher gain in whole-body BMD in rats compared with those receiving casein or soy protein alone (Figure 1).

### 4.3. Femoral and Fourth Lumbar BMD and BMC

The right femoral BMC and BMD were significantly reduced in ovariectomized rats in comparison with intact rats (Table 2). Animals on the soy + FOS treatment had femoral BMC similar to the sham animals and significantly higher than the Ovx rats receiving the casein-based diet. Dried plum in the diet (Ovx + soy + plum or Ovx + soy + plum + FOS) increased femoral BMC to the levels of the sham animals but still similar to the Ovx rats receiving the casein-based diet. In Ovx rats, in terms of BMD, addition of any of the bioactive compounds to a soy protein-based diet resulted in a significantly higher femoral BMD compared with casein, albeit not to the level of the intact rats (Table 2). The Ovx-induced reduction in total mineral content (% ash) of the right femur was modulated by the addition of FOS (Ovx + soy + FOS) or dried plum alone to soy protein-based diet but not their combination (Ovx + soy + plum + FOS).

Similar to the femur, ovariectomy significantly reduced BMC, BMD and % ash of the fourth lumbar, a site mostly composed of trabecular bone (Table 2). In Ovx rats, the decrease in these parameters due to ovariectomy was modulated by soy-protein diet with added bioactive component but not quite to the level of sham rats. Ovariectomized rats fed the combination of soy, dried plum and FOS (Ovx + soy + plum + FOS) have the highest fourth lumbar BMD, but again, not quite to the level of sham rats.

### 4.4. Biomechanical Properties of the Left Femur

**Figure 2:**
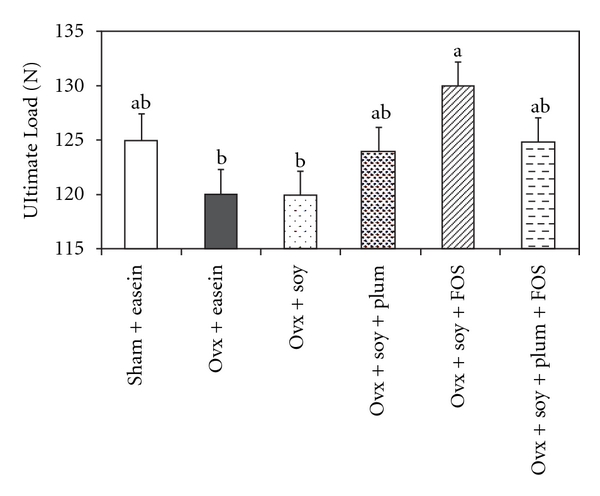
Effects of ovariectomy (Ovx) and various dietary treatments on ultimate load of the left femur. Bars represent mean + SE; *n* = 12 rats per group. Bars with different letters are significantly (*P* < .05) different from each other.

In Ovx rats, ovariectomy somewhat reduced ultimate load, an indicator of the material properties of bone, compared with sham rats (Figure 2). However, femurs of ovariectomized rats fed a soy protein-based diet with added FOS (Ovx + soy + FOS) withstood the greatest loads. There were no significant changes in other biomechanical properties such as yield load, stiffness, ultimate stress, yield stress and modulus of elasticity due to ovariectomy or dietary interventions (data not shown).

### 4.5. Histomorphometric Analyses of the Tibia

Ovariectomy caused an increase in trabecular thickness (Tb Th), trabecular separation (Tb Sp) and mineral surface as percent of bone surface (MS/BS) (Table 3). Any of the diets containing FOS slightly improved Tb Sp to the sham level but still similar to the Ovx control rats. Ovariectomized rats receiving diets containing the combination of soy with dried plum (Ovx + soy + plum) had Tb Th values that were moderately brought down to levels similar to those of intact rats, yet still high enough to also be similar to those observed in the Ovx group. Diets containing the combination of soy with plum and FOS (Ovx + soy + plum + FOS) reduced MS/BS to the sham level. Trabecular number was decreased due to ovariectomy and dietary treatments did not prevent the ovariectomy-induced decrease on this parameter.

### 4.6. Serum and Urine Analyses

As expected, ovariectomy significantly reduced serum levels of E_2_, thus confirming the success of the surgery (Table 4). Ovariectomy slightly increased serum ALP activity and soy protein in combination with dried plum (Ovx + soy + plum) or FOS (Ovx + soy + FOS) further increased serum ALP activity. Ovariectomized rats receiving the casein-based diet had significantly higher excretion of urinary Dpd, a specific marker of bone resorption, compared with intact rats. This increase in urinary Dpd excretion was ameliorated to levels similar to those of sham rats by all of the soy protein-based diets except the combination of soy with dried plum (Ovx + soy + plum), which produced an intermediate effect. This finding indicated that the combinations of these dietary components increased the ovariectomy-induced rate of bone formation while reducing the ovariectomy-induced increase in bone resorption.

## 5. Discussion

Inhibiting further bone loss and reducing the risk of fracture are hallmarks for an effective antiosteoporotic agent. Although there are a number of conventional medicines for preventing and/or treating osteoporosis, these medicines are not devoid of side effects and are also costly. In addition, the long-term patient adherence rates are low. The purpose of our research is to find functional foods that can act as such agents by preventing bone loss or promoting bone restoration. The present study was designed to determine if combining soy protein with FOS, dried plum or both was more effective at restoring bone loss as a result of ovarian hormone deficiency.

The findings of this study show that while a soy protein-based diet alone was not sufficient to reverse the loss in whole-body, femoral and fourth lumbar BMD due to ovariectomy, the combination of soy protein with other bioactive components such as FOS and dried plum was able to improve BMD of osteopenic Ovx rats. Soy protein alone offers minimal beneficial effect on BMD while FOS and dried plum provide additive effects in terms of bone density in this animal model. In general, the effect of soy protein on bone is controversial and inconclusive. Soy protein has been shown to have no significant effect [10, 17–21] or a slight increase [7, 8, 11, 22, 23] in BMD in both animal studies and small clinical trials. Its efficacy however is awaiting confirmation via large clinical trials that are currently underway.

The higher whole-body BMD in diets supplemented with FOS (Ovx + soy + FOS, Ovx + soy + FOS + plum) suggests that FOS, a mixture of indigestible and fermentable sugars, has a bone reversal effect. FOS has been shown to increase absorption of minerals from the colon [24–26] and increase calcium balance and bone mineralization, as well as decrease bone turnover rate in Ovx rats [27] and mice [28]. It has been reported that FOS can maximize the bone sparing effects of soy–isoflavone enriched diet in Ovx rats by modulating the bioavailability of isoflavones [27, 29]. In the present study, we observed that the rats receiving FOS plus soy had higher levels of serum ALP and femurs that withstood greatest load strain. Increased bone density may lead to enhanced bone strength, reducing the risk of fracture, which is evident in the soy plus FOS diet due to greater whole-body BMD and greatest load strain tolerance.

Combining FOS and dried plum significantly improved whole-body BMD, femoral BMC and BMD and fourth lumbar BMD. The bone protective effect of the combination of soy with dried plum and FOS may be due to its ability to decrease bone resorption, as shown by lower urinary Dpd excretion. The dietary bioactive components caused a reduction in bone resorption and maintained the increased rates of bone formation due to ovariectomy, thereby resulting in a net bone gain in bone density. It is interesting to note that whole-body BMD improvements were similar in the soy protein diets combined with FOS alone (Ovx + soy + FOS) and with both FOS and dried plum (Ovx + soy + plum + FOS); however, there seems to be distinct differences in the ability to improve bone health at different sites. Soy plus FOS seemed to improve BMD the greatest at the right femur while greatest improvement in fourth lumbar BMD was seen in the soy plus dried plum and FOS group.

In the present study, deterioration of Ovx-induced trabecular microarchitecture was restored by adding dried plum to a soy diet. Trabecular thickness increase due to Ovx has been suggested to be a compensation for lost trabecular connectivity [30]. Soy plus dried plum decreased Tb Th to sham levels, ameliorating the deterioration, while soy + FOS + plum decreased MS/BS to sham level. In addition, any diet supplemented with FOS was able to restore bone volume as percentage of tissue volume (BV/TV) to the level of sham but was not significantly different from Ovx, suggesting that this diet can prevent decline associated with ovarian hormone deficiency. Histomorphometric changes promoting skeletal strength via diet may aid in reducing the incidence of osteoporotic fracture naturally and offer a promising alternative to those women who do not seek conventional medicinal treatments.

Animals on a soy + FOS + plum diet showed an 8% increase in fourth lumbar BMD and a 5% increase in both whole-body and right femur BMD. These percent increases are considered clinically relevant when results are compared with studies using approved medications for treating osteoporosis. For instance, Ito et al. [31] showed that orally administered alendronate improved lumbar BMD by 7.31% after 10 weeks of treatment in 1-year-old rats. Furthermore, Bourrin et al. [32] showed only a 4.2% increase in tibial BMD. In the present study, treatment of soy+FOS+plum additionally showed a 9.5% increase in Tb Th and 36% increase in MS/BS compared with ovx animals. Our results suggest that combining dried plum and FOS to a soy-based diet showed results that are at least equivalent and may surpass those of conventional medications.

The results from this study are in agreement with those from our previous observations [12–14] that suggest the efficacy of dried plum exceeds that of soy protein and its isoflavones. Our earlier animal data indicate that dried plum not only protects against [12, 14] but more importantly reverses bone loss in two separate models of osteopenia [13, 33, 34]. Our initial study [12] using Ovx rats indicated that dried plum prevented the ovariectomy-induced reduction in BMD of the femur and lumbar vertebra. In another study in which Ovx rats were allowed to lose bone before the initiation of treatment, dried plum was able to restore BMD and bone strength to the level of sham-operated rats [13]. Dried plum was also efficacious in restoring the loss in trabecular microarchitectural properties of the tibia and the lumbar vertebra. Furthermore, we have also shown that dried plum enhanced bone recovery during reambulation following skeletal unloading and had comparable effects to PTH [33, 34]. Our positive animal findings on the effect of dried plum on bone were confirmed by our short-term clinical trial [15] in which dried plum supplementation significantly increased indices of bone formation (ALP, bone-specific ALP levels and IGF-I) in postmenopausal women while reducing bone resorption.

Relevant to the present study, it may be worth investigating the mechanisms underlying the additive effects of soy with the different bioactive compounds to provide an explanation for these findings. Adding dried plum and/or FOS to a soy-based diet can offer improvements beyond that of soy alone and results suggest that some of the combinations may affect different areas with varying degrees. Therefore, it is necessary to investigate if these bioactive compounds either together or independently can also restore bone mass in postmenopausal women. These positive effects of dried plum and FOS on biomarkers of bone metabolism have resulted in an improvement of BMD in this animal model. FOS and dried plum combined with soy in the diet provides a valuable alternative to conventional osteoporosis therapies due to their anti-resorptive and anabolic properties.

## Figures and Tables

**Table 1:** Effects of ovariectomy (Ovx) and various dietary treatments on food intake and body and uterine weights.

Parameters	Sham-casein	Ovx + casein	Ovx + soy	Ovx + soy + plum	Ovx + soy+ FOS	Ovx + soy + plum + FOS
Food intake (g/d)	14.1 ± 0.4	14.0 ± 0.3	13.9 ± 0.3	13.9 ± 0.3	14.1 ± 0.4	14.2 ± 0.3
*Body weights* (g)						
Initial	231 ± 1	231 ± 1	232 ± 1	231 ± 1	230 ± 1	232 ± 1
Final	281 ± 4^b^	317 ± 4^a^	315 ± 4^a^	319 ± 4^a^	327 ± 4^a^	316 ± 4^a^
Uterine weight (mg)	606 ± 33^a^	185 ± 31^b^	182 ± 30^b^	169 ± 30^b^	132 ± 31^b^	141 ± 31^b^

Values are mean ± SE; *n* = 12. Within a row, values that do not share the same superscript letters are significantly (*P* < .05) different from each other.

**Table 2:** Effects of ovariectomy (Ovx) and various dietary treatments on whole body, right femur and fourth lumbar BMD and BMC.

Parameters	Sham-casein	Ovx + casein	Ovx + soy	Ovx + soy + plum	Ovx + soy + FOS	Ovx + soy + plum + FOS
Whole body						
BMC (g)	10.268 ± 0.164	10.397 ± 0.164	10.297 ± 0.157	10.648 ± 0.157	10.779 ± 0.164	10.625 ± 0.164
BMD (mg/cm^2^)	172.4 ± 1.4^a^	159.8 ± 1.4^c^	161.4 ± 1.4^c^	165.2 ± 1.4^b,c^	167.2 ± 1.4^b^	167.5 ± 1.4^b^
Right femur						
BMC (mg)	473.9 ± 8.8^a^	435.0 ± 8.8^c^	447.5 ± 8.4^b,c^	450.1 ± 8.4^a,b,c^	464.6 ± 8.8^a,b^	454.9 ± 6.4^a,b,c^
BMD (mg/cm^2^)	250.7 ± 2.5^a^	226.2 ± 2.5^c^	232.9 ± 2.4^b,c^	235.5 ± 2.4^b^	238.2 ± 2.5^b^	237.5 ± 2.5^b^
Ash (%)	68.2 ± 0.5^a^	66.6 ± 0.5^b^	65.9 ± 0.5^b^	66.8 ± 0.5^a,b^	67.2 ± 0.5^a,b^	66.3 ± 0.5^b^
Fourth Lumbar						
BMC (mg)	152.4 ± 3.7^a^	128.4 ± 3.7^d^	131.5 ± 3.6^c,d^	138.5 ± 3.6^b,c,d^	142.0 ± 3.7^b^	140.7 ± 3.7^b,c^
BMD (mg/cm^2^)	246.2 ± 3.8^a^	215.0 ± 3.8^d^	219.7 ± 3.7^c,d^	222.5 ± 3.7^c,d^	228.1 ± 3.8^b,c^	232.3 ± 3.8^b^
Ash (%)	63.3 ± 0.6^a^	56.3 ± 0.6^c^	57.0 ± 0.6^b,c^	58.5 ± 0.6^b^	58.5 ± 0.6^b^	58.7 ± 0.6^b^

Values are mean ± SE; *n* = 12. Within a row, values that do not share the same superscript letters are significantly (*P* < .05) different from each other.

**Table 3:** Effects of ovariectomy (Ovx) and various dietary treatments on histomorphometric parameters of the left proximal tibia.

Parameters	Sham-casein	Ovx + casein	Ovx + soy	Ovx + soy + plum	Ovx + soy + FOS	Ovx + soy + plum + FOS
BV/TV (%)	11.9 ± 1.9^a^	8.1 ± 1.9^a,b^	6.7 ± 1.9^a,b^	5.2 ± 1.9^b^	8.3 ± 1.9^a,b^	9.9 ± 1.9^a,b^
Tb Th (*μ*m)	46 ± 5^c^	63 ± 5^a,b^	58 ± 5^a,b,c^	53 ± 5^b,c^	61 ± 5^a,b^	69 ± 5^a^
Tb N (*n*)	2.46 ± 0.27^a^	1.09 ± 0.27^b^	1.19 ± 0.27^b^	0.99 ± 0.27^b^	1.26 ± 0.27^b^	1.42 ± 0.27^b^
Tb Sp (*μ*m)	441 ± 198^b^	1359 ± 198^a^	1032 ± 198^a^	1125 ± 198^a^	959 ± 198^a,b^	942 ± 198^a,b^
MS/BS (%)	2.2 ± 2.0^b^	10.2 ± 2.0^a^	10.1 ± 2.0^a^	8.9 ± 2.0^a^	12.1 ± 2.0^a^	6.5 ± 2.0^ab^

Values are mean ± SE; *n* = 6. Within a row, values that do not share the same superscript letters are significantly (*P* < .05) different from each other. BV/TV = bone volume as percentage of tissue volume; Tb N = trabecular number.

**Table 4:** Effects of ovariectomy (Ovx) and various dietary treatments on serum and urinary parameters.

Parameters	Sham-casein	Ovx + casein	Ovx + soy	Ovx + soy + plum	Ovx + soy + FOS	Ovx + soy + plum + FOS
Serum						
17-*β* estradiol (pg/mL)	21.9 ± 5.4^a^	7.2 ± 1.9^b^	6.4 ± 1.5^b^	5.3 ± 1.0^b^	3.8 ± 0.4^b^	4.9 ± 0.9^b^
ALP (U/L)	41.7 ± 4.3^b^	51.4 ± 4.3^a,b^	49.8 ± 4.1^a,b^	55.3 ± 4.1^a^	60.9 ± 4.4^a^	52.7 ± 4.3^a,b^
Urine Dpd	39.9 ± 8.9^b^	98.9 ± 25.8^a^	57.1 ± 3.7^b^	66.9 ± 17.8^a,b^	48.0 ± 3.2^b^	54.2 ± 3. 1^b^
(nmol/mmol creatinine)						

Values are mean ± SE; *n* = 12. Within a row, values that do not share the same superscript letters are significantly (*P* < .05) different from each other.
